# Gonorrhea in the Male

**Published:** 1911-09

**Authors:** 


**Gonorrhea in the Male.** A Practical Guide to its Treatment. By **Abr. L. Wolbarst,** M.D., Consulting Genitourinary Surgeon, Central Islip State Hospital. New York: International Journal of Surgery Co. 1911. (Cloth, $1.00.).

Wohlbarst belives that much harm is done by the overzealous treatment of acute gonorrhoea in vogue at the present day. He considers the irrigation method to be efficacious but dangerous in unskilled hands. Injections should be administered by the surgeon personally until the acute symptoms have subsided and internal medication is heartily endorsed.

The author claims that under his method of treatment complications except prostatitis are reduced to a minimum. The technique of meatomy is discussed in detail especial stress being laid upon the necessity of dividing the fold of Guerin, when it encroaches upon the urethral lumen.

Some of Wohlbarst's views as to the indications for urethrotomy are at variance with those prevalent among the majority of urologists. This book is especially to be commended for emphasising the fact that the urethra is a sensitive organ and not merely an elastic tube. C. W. B.

